# Influenza Vaccination Results in Differential Hemagglutinin Stalk-Specific Fc-Mediated Functions in Individuals Living With or Without HIV

**DOI:** 10.3389/fimmu.2022.873191

**Published:** 2022-04-19

**Authors:** Boitumelo M. Motsoeneng, Nisha Dhar, Marta C. Nunes, Florian Krammer, Shabir A. Madhi, Penny L. Moore, Simone I. Richardson

**Affiliations:** ^1^ HIV Virology Section, Centre for HIV and STIs, National Institute for Communicable Diseases of The National Health Laboratory Services, Johannesburg, South Africa; ^2^ South African Medical Research Council Antibody Immunity Research Unit, Faculty of Health Sciences, University of the Witwatersrand, Johannesburg, South Africa; ^3^ South African Medical Research Council Vaccines and Infectious Diseases Analytics Research Unit, Faculty of Health Sciences, University of the Witwatersrand, Johannesburg, South Africa; ^4^ Department of Science and Innovation/National Research Foundation, South African Research Chair Initiative in Vaccine Preventable Diseases Unit, Faculty of Health Sciences, University of the Witwatersrand, Johannesburg, South Africa; ^5^ Department of Microbiology, Icahn School of Medicine at Mount Sinai, New York, NY, United States; ^6^ Department of Pathology, Molecular and Cell based Medicine, Icahn School of Medicine at Mount Sinai, New York, NY, United States; ^7^ African Leadership in Vaccinology Expertise (ALIVE), Faculty of Health Sciences, University of the Witwatersrand, Johannesburg, South Africa; ^8^ Centre for the AIDS Programme of Research in South Africa (CAPRISA), University of KwaZulu Natal, Durban, South Africa; ^9^ Institute of Infectious Disease and Molecular Medicine, University of Cape Town, Cape Town, South Africa

**Keywords:** influenza vaccination, Fc effector functions, HIV co-infection, hemagglutinin stalk antibodies, antibody-dependent cellular phagocytosis (ADCP), antibody-dependent complement deposition (ADCD), antibody- dependent cellular cytotoxicity (ADCC)

## Abstract

Influenza virus hemagglutinin (HA) stalk-specific antibodies have been shown to potently induce Fc-mediated effector functions which are important in protection from disease. In placebo-controlled maternal influenza (MatFlu) vaccination trials of pregnant women living with or without HIV, reduced risk of influenza illness was associated with high HA stalk antibody titers following trivalent inactivated vaccination (TIV). However, the mechanisms of immunity conferred by the HA stalk antibodies were not well understood. Here, we investigated HA stalk-specific Fc effector functions including antibody-dependent cellular phagocytosis (ADCP), antibody-dependent cellular cytotoxicity (ADCC), antibody-dependent complement deposition (ADCD), and FcγRIIa and FcγRIIIa binding in response to seasonal influenza vaccination. These were measured pre- and 1-month post-vaccination in 141 HIV-uninfected women (67 TIV and 74 placebo recipients) and 119 women living with HIV (WLWH; 66 TIV and 53 placebo recipients). In contrast to HIV-uninfected women, where HA stalk-specific ADCP and FcγRIIa binding were significantly boosted, WLWH showed no increase in response to vaccination. HA stalk-specific ADCC potential and FcγRIIIa binding were not boosted regardless of HIV status but were higher in WLWH compared with HIV-uninfected women prior to vaccination. HA stalk-specific ADCD was significantly increased by vaccination in all women, but was significantly lower in the WLWH both pre- and post- vaccination. Co-ordination between HA stalk-specific ADCP and ADCD in WLWH was improved by vaccination. Fc polyfunctionality was enhanced by vaccination in HIV-uninfected women and driven by the HA stalk antibody titers. However, in the WLWH, higher pre-vaccination Fc polyfunctionality was maintained post-vaccination but was decoupled from titer. Overall, we showed differential regulation of Fc effector HA stalk responses, suggesting that HIV infection results in unique humoral immunity in response to influenza vaccination, with relevance for future strategies that aim to target the HA stalk in this population.

## Introduction

Seasonal influenza epidemics cause over 56,000 hospitalizations and 11,000 deaths annually in South Africa (1). Immunocompromised individuals such as pregnant women and people living with HIV (PLWH) are especially burdened with severe respiratory disease. Therefore seasonal trivalent inactivated influenza vaccines (TIV) are recommended for these high-risk individuals and have been shown to have a significant impact on public health (2). Whilst TIV efficacy has been confirmed in PLWH, vaccine immunogenicity was suboptimal in these individuals (3–8). Therefore, there is a need to further understand the mechanisms of immunity in PLWH, following seasonal influenza vaccination.

Humoral immune responses elicited by TIVs primarily target the viral hemagglutinin (HA), which is composed of a head and stalk domain. The ability of HA head-specific antibodies to neutralize influenza virus, detected using hemagglutination inhibition (HAI) assays, is considered a relative correlate of protection (9). However, the HA head domain continuously undergoes antigenic drift, allowing escape from HA head-specific antibodies induced from previous viral exposures and vaccinations (10). The immuno-subdominant, but conserved HA stalk domain is a target for the development of broadly protective influenza vaccines (11). In addition to having neutralizing activity, HA stalk antibodies confer protection through Fc-FcγR interactions (12).

Fc effector functions have been associated with protection against influenza virus infection, in experimental challenge models and after vaccination (13–17). Through the interaction of the antibody Fc region with cell surface Fc receptors or complement proteins, cytotoxic functions such as antibody-dependent cellular phagocytosis (ADCP), cellular cytotoxicity (ADCC) and complement deposition (ADCD) occur. In animal models, ADCP, ADCC and ADCD have been associated with protection against infection (18–21). In humans, seasonal influenza vaccination enhances cross-reactive ADCC and ADCP antibodies directed to the HA in healthy individuals and high-risk groups, such as older adults and PLWH (22–24). In these studies, TIV boosted Fc effector functions when head-specific HAI responses were low, highlighting the potential of HA stalk antibodies and their cytotoxic functions for protection in immunocompromised individuals.

In general, PLWH are at a higher risk of deaths associated with severe influenza disease (25). B-cell impairments and reduced HAI antibody levels in response to seasonal TIV have been observed in this group, with pregnancy further increasing susceptibility to severe influenza virus infections (26–30). PLWH on antiretroviral treatment (ART) have lower HAI responses in comparison to HIV-uninfected individuals even when TIV doses were increased or a second dose was administered (5, 6, 8, 31). However, studies focusing on the HA stalk are limited and detailed antibody responses and mechanisms of immunogenicity in this high-risk group are not well understood.

In two randomized, double-blind, placebo-controlled maternal influenza (MatFlu) vaccination trials, lower HAI titers were observed in pregnant women living with HIV (WLWH) compared with pregnant women living without HIV (6). However, the vaccine efficacy against confirmed influenza illness in WLWH trended to being higher (70.6%) than in HIV-uninfected women (54.4%) (6). This indicated that antibody responses to epitopes that lie beyond the HA head may be important in protection in this high-risk group. A follow up study showed that HA stalk antibody titers were associated with reduced risk of influenza virus infection (32). Given that HA stalk-specific antibodies mediate potent Fc effector functions and are known to be protective, we explored the Fc-mediated functions of HA stalk-specific antibodies in this cohort of WLWH.

In this study we observed higher HA stalk-specific *in vitro* ADCP and ADCC as measured by a reporter assay in WLWH prior to vaccination in comparison with HIV-uninfected women. ADCC potential directed at the HA stalk was not boosted regardless of HIV status, whereas ADCP was enhanced only in HIV-uninfected women. In both groups HA stalk-specific ADCD was increased although this function is compromised in WLWH. We also observed differences in the coordination of Fc effector functions post-vaccination. In WLWH the association between HA stalk-specific ADCP and ADCD was enhanced but in the HIV-uninfected women these functions were associated with HA stalk-specific ADCC potential. We also show that Fc polyfunctionality was higher in WLWH prior to vaccination and was not further enhanced, whereas in HIV-uninfected women it was improved. This study suggests that seasonal influenza vaccination may confer protection through different HA stalk targeted mechanisms which may include Fc effector functions for WLWH and HIV-uninfected women.

## Materials and Methods

### Ethics Statement

The 2011 maternal influenza (MatFlu) vaccination trials (approval numbers: 101106 and 101107) and this sub-study (approval number: M200444) were approved by the Human Research Ethics Committee of the University of the Witwatersrand. All study participants provided written informed consent to have their stored samples used for future studies. All healthy donors in this study provided written informed consent to obtain plasma, isolate IgG and have their samples stored for future use.

### Influenza Vaccination Cohort

The two randomized, double-blind, placebo-controlled MatFlu vaccination trials conducted in Soweto, South Africa have been previously described (6). Briefly, women, all of whom were pregnant, between the ages of 18-38 years were stratified according to their HIV status and randomly assigned (1:1) to the placebo or vaccine groups. The trivalent inactivated vaccine (TIV) used in the trials was Vaxigrip, which contained 15 µg each of A/California/7/2009 (A/H1N1/pdm09), A/Victoria/210/2009 (A/H3N2), and a B/Brisbane/60/2008-like virus (B/Victoria) as recommended by the World Health Organization for the Southern Hemisphere in 2011. Active surveillance for respiratory illness and PCR-confirmed influenza-illness was performed. In this study, plasma samples from a sub-set of HIV-uninfected women (74 placebo-recipients and 67 vaccinees) and WLWH (53 placebo-recipients and 66 vaccinees), collected prior to vaccination and one-month post-vaccination, were tested. Data from participants with PCR-confirmed influenza-illness were excluded from this study.

### Protein Expression and Pooled Plasma/IgG Preparation

A chimeric recombinant hemagglutinin (cHA) protein, composed of an H6 head and an H1 stalk (cH6/1) was produced as previously described (33). For use as a positive control in all the Fc assays, plasma was pooled from 5 healthy donors with high HA stalk IgG titers. From this pooled plasma IgG was isolated using Protein G (Pierce Biotechnology), according to the manufacturer’s instructions and confirmed by IgG enzyme linked immunosorbent assay (ELISA). A cross-reactive HA stalk antibody CR9114 that mediates potent Fc effector function was expressed as a positive control (34). For antibody expression, plasmids encoding heavy or light chain genes were co-transfected into HEK293F cells with PEI-MAX 40,000 (Polysciences) head-to-head. Cells were cultured for six days in 293F Freestyle media at 37°C, 10% CO_2_, then harvested supernatants were filtered and purified using Protein G (Thermo Scientific). Antibody concentration was quantified by nanodrop using sequence-specific extinction coefficients as determined by ProtParam (ExPASy) and confirmed by ELISA.

### Hemagglutinin Inhibition (HAI) Assay

Modified HAI assays were previously performed (6, 35). Briefly, plasma samples instead of serum were treated with receptor-destroying enzyme (RDE) from *Vibrio cholera* (Denka-Seiken). Then these were diluted 1:10 in saline and subsequent serial 2-fold dilutions of the plasma were used in a standard HAI assay using 4 hemagglutinating units of the antigen and 0.75% turkey red blood cells. Plasma samples with titers ≥10 were considered indicative of immune responses. The antigen used in the assays was A/H1N1/pdm09.

### Hemagglutinin Stalk (H1/stalk) IgG ELISA

H1/stalk IgG ELISAs were performed as previously described (32). The cH6/1 recombinant protein described above was utilized and binding against this protein measures antibodies against the H1 stalk domain. Plates were coated with cH6/1 diluted to 2 µg/ml overnight at 4°C. Plates were washed and subsequently blocked with 3% fetal bovine serum (Biowest), 0.5% non-fat dry milk powder (Bio-Rad) for 2 hours at room temperature (RT). After washing, plasma samples were added to the plate diluted to a starting concentration of 1:40, followed by two-fold serial dilution and incubated for 2 hours at RT. Following a wash, anti-human IgG (Fab specific)-peroxidase antibody (Sigma, USA) diluted to 1:3000 in blocking solution was added to the plate. After 1-hour incubation at RT, plates were washed and developed using SigmaFast *o*-phenylenediamine dichloride (OPD) (Sigma, USA) for 10 min at RT. The reaction was stopped by adding 3M HCl and the optical density (OD) was measured at 490 nm. The antibody concentration was quantified against the standard curve included on each plate that consisted of polyvalent human normal immunoglobulin (Polygam) (National Bioproducts Institute, South Africa) with an assigned arbitrary value of 1000 arbitrary units (AU)/ml.

### Antibody-Dependent Cellular Phagocytosis (ADCP)

The ADCP assay was performed as previously described (36). Briefly The EZ-Link Sulfo-NHS-LC-Biotin kit (Thermo Scientific) was used to biotinylate cH6/1, which was coated onto fluorescent neutravidin beads (Invitrogen). The coated beads were incubated for 2 hours with mAbs at a final concentration of 50 µg/ml or a 1:100 dilution of plasma sample, prior to overnight incubation with a monocytic cell line, THP-1 cells. The THP-1 cells were obtained from the NIH AIDS Reagent Program and cultured at 37°C, 5% CO_2_ in Roswell Park Memorial Institute (RPMI) 1640 media supplemented with 10% fetal bovine serum (FBS), 100 units/ml Penicillin and 100 ug/ml Streptomycin (Gibco), referred to as R10, and not allowed to exceed 4 x 10^5^ cells/ml. This assay was completed on a FACS Aria II (BD Biosciences). Phagocytic scores were calculated as the geometric mean fluorescent intensity (MFI) of the beads multiplied by the percentage bead uptake minus the no antibody control as background. For this and all functional Fc assays, pooled plasma from 5 healthy donors with previous exposures to influenza, screened for H1 stalk antibodies and CR9114 were used as positive controls and Palivizumab (RSV mAb; MedImmune, LLC) and VRC01 (In house HIV mAb) were used as negative controls. In order to normalize across plates and runs, the pooled plasma positive scores were averaged, divided by the pooled plasma score per plate and this normalizing factor multiplied across the scores.

### Antibody-Dependent Cellular Cytotoxicity (ADCC) Reporter Assay

The ability of plasma antibodies to cross-link cH6/1 HA stalk antigen and activate FcγRIIIa on Jurkat-Lucia™ NFAT-CD16 cells (*In vivo*gen) was measured as a proxy for ADCC or cell lysis. These cells *In vivo* were cultured according to the manufacturer’s instructions. Adapted from elsewhere (37), high-binding 96 well plates were coated with 1 μg/mL cH6/1 and incubated at 4°C overnight. Plates were then washed with phosphate buffered saline (PBS) and blocked at room temperature for 1 hour with 2.5% bovine serum albumin (BSA)/PBS. After washing, mAbs at a starting concentration of 1 mg/ml or a 1:10 dilution of plasma sample was added and incubated for 1 hour at 37°C. Subsequently, 2x 10^5^ cells/well in R10 were added and incubated for 24 hours at 37°C, 5% CO_2_. Then 25 µl of supernatant was transferred to a white 96-well plate with 75 µl of reconstituted QUANTI-Luc secreted luciferase and read immediately on a Victor 3 luminometer (PerkinElmer) with 1s integration time. Relative light units (RLU) of a no antibody control were subtracted as background. The pooled plasma and CR9114 were used as positive controls and Palivizumab and VRC01 were used as negative controls. The reported values are the mean of three kinetic reads taken at 0, 2.5, and 5 min. To induce the transgene 1x cell stimulation cocktail (Thermo Scientific) and 2 μg/ml ionomycin in R10 was added as a positive control to confirm sufficient expression of the CD16 Fc receptor. ADCC RLUs were normalised across plates and runs, by averaging the pooled plasma RLUs and multiplying the RLUs across the samples with the normalizing factor.

### Antibody-Dependent Complement Deposition (ADCD)

ADCD was measured using a previously described high-throughput bead-based assay (38). Biotinylated cH6/1 was coated 1:1 onto fluorescent neutravidin beads (Invitrogen) for 2 hours at 37°C. The antigen-coated beads were incubated with a 1:10 plasma sample dilution or mAbs at a starting concentration of 100 μg/ml for 2 hours and incubated with guinea pig complement diluted 1 in 50 with gelatin/veronal buffer for 15 minutes at 37°C. Beads were washed in PBS and stained with anti-guinea pig C3b-FITC, fixed and interrogated on a FACS Aria II (BD Biosciences). Complement deposition scores were calculated as the percentage of C3b-FITC positive beads multiplied by the geometric MFI of FITC in this population minus the no antibody or heat inactivated controls. The pooled plasma and CR9114 were used as positive controls and Palivizumab and VRC01 were used as negative controls. ADCD scores were normalised between plates and runs, by dividing the ADCD score of the pooled plasma by the average across the plates and multiplying the scores with this normalizing factor.

### Dimeric Fc Gamma Receptor Binding ELISAs

High-binding 96 well ELISA plates were coated with 1 ug/ml cH6/1 in PBS overnight at 4°C. Three wells on each plate were directly coated with 5 ug/ml IgG, isolated from healthy donors, signals from these wells were used to normalize the FcR activity of the plasma samples and pooled plasma was used as a positive control. Plates were washed with PBS and blocked with PBS/1 mM ethylenediaminetetraacetic acid (EDTA)/1% BSA for 1 hour at 37°C. Plates were then washed and incubated with 1:10 diluted plasma for 1 hour at 37°C and then with 0.2 ug/ml of biotinylated FcγRIIa dimer or 0.1 ug/ml of biotinylated FcγRIIIa dimer for 1 hour at 37°C. The dimeric FcγRs were provided by Prof. Mark Hogarth from the Burnet Institute, Melbourne, Australia (39). Subsequently, a 1:10000 dilution of Pierce high-sensitivity streptavidin-horseradish peroxidase (Thermo Scientific) was added for a final incubation of 1 hour at 37°C. Lastly, TMB (3,3,5,5-tetramethylbenzidine) substrate (Sigma-Aldrich) was added, colour development was stopped with 1 M sulfuric acid and absorbance read at 450 nm.

### Statistical Analysis

Data were analyzed in Prism (v9; GraphPad Software Inc., San Diego, CA, USA). Non-parametric tests were used for all comparisons. The Mann-Whitney and Wilcoxon tests were used for unmatched and paired samples, respectively. Fc polyfunctionality Z-scores were calculated by subtracting the mean of the Fc function from the individual value and divided by the standard deviation of the mean and then adding all the Z-scores for each function per individual. The proportions of responders and non-responders in each group and the proportions of participants with high or low Fc polyfunctionality Z-scores were compared by Fisher’s exact tests. All correlations reported are non-parametric Spearman’s correlations. *P* values less than 0.05 were considered statistically significant.

## Results

### Women Living With HIV (WLWH) Have Lower HAI and H1 Stalk Antibody Titers in Response to Seasonal Influenza Vaccination

**Figure 1:**
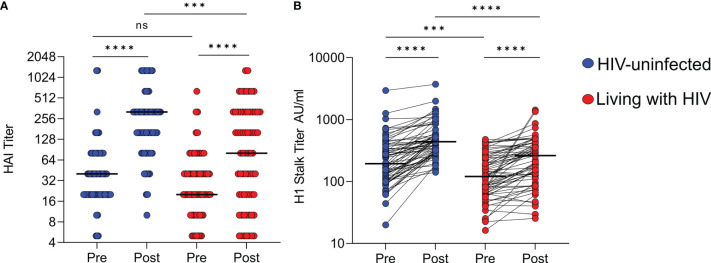
A/H1N1 HAI and H1 stalk antibody responses amongst vaccinated women. **(A)** Pre-vaccination and 1-month post-vaccination hemagglutination inhibition (HAI) titers against A/H1N1 and **(B)** H1 stalk titers by ELISA, with HIV-uninfected participants (n=67), shown in blue and participants living with HIV (n=66), shown in red. The lines represent the median. Wilcoxon matched-pairs signed rank test used to compare pre- and post-vaccination titers. Mann Whitney U test used to compare responses between vaccine groups. Significant associations shown as ****p < 0.0001; ***p < 0.001; ns,not significant.

In our previous study, HAI titers were significantly boosted by vaccination regardless of HIV status, but WLWH showed significantly lower HAI titers post-vaccination compared to HIV-uninfected women (6). We confirmed this finding in a subset of plasma samples from pregnant vaccinated participants from the MatFlu cohort, including 67 HIV-uninfected women and 66 WLWH, matched in age with a median of 25 years (range 18-39) and 27 years (range 18-38) respectively, and excluded all PCR-confirmed influenza virus infection cases. Plasma samples were collected pre-vaccination and post-vaccination at a median of 31 days (range 28-33) for HIV-uninfected and 30 days (range 28-31) for WLWH (**Supplementary Table 1**). Post-vaccination HAI titers were significantly lower in WLWH compared with HIV-uninfected women (medians 80 vs. 320 A/H1N1 HAI titer) (**Figure 1A**), coupled with decreased boosting levels following vaccination (median fold change 1.5 vs. 4) (**Supplementary Figure 1A**). In these samples, we also confirmed that post-vaccination H1 stalk antibody titers were significantly lower in WLWH compared with HIV-uninfected women (medians 259.3 vs. 433.5 AU/ml) (**Figure 1B**). This was despite vaccine boosting H1 stalk antibody titers in both groups, which was consistent with the parent study (32). Furthermore, we also confirmed that in contrast to HAI titers, the HIV-uninfected women and WLWH had more comparable fold increases in the H1 stalk antibody titer boosting (median fold change 1.9 vs. 1.7) (**Supplementary Figure 1B**). As expected, no changes in HAI and H1 stalk antibody titers amongst placebo recipients living with or without HIV were observed (**Supplementary Figure 2**).

### Women Living With HIV Have Higher Pre-Vaccination ADCP and ADCC Reporter Activity, but These Are Not Boosted by Seasonal TIV

**Figure 2:**
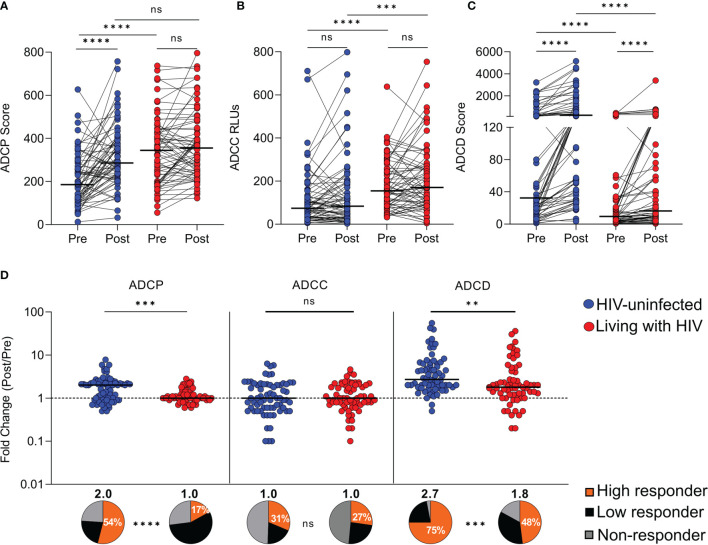
Differential boosting of HA stalk-specific Fc-mediated antibody functions amongst vaccinated women. Pre-vaccination and 1-month post-vaccination H1 stalk-specific **(A)** antibody-dependent cellular phagocytosis (ADCP), **(B)** antibody-dependent cellular cytotoxicity (ADCC) or FcγRIIIa activation and **(C)** antibody-dependent complement deposition (ADCD) are shown where HIV-uninfected participants (n=67), shown in blue and participants living with HIV (n=66), shown in red.**(D)** The fold increases for each of the functions and the proportion (%) of high responders that exceed a 2-fold increase in Fc activity are shown in orange. Low responders, who show reduced enhancement that does not reach a 2-fold increase are shown in black, whilst those not boosted (non-responders) are shown in grey. The lines represent the median. Wilcoxon matched-pairs signed rank tests were used to compare pre-vaccination and post-vaccination functional scores within groups. Mann Whitney U test was used to compare functional scores between the vaccine groups. Fischer’s exact tests were used to compare the proportions of high responders. Significant associations are shown as ****p < 0.0001; ***p < 0.001; **p < 0.01; ns,not significant.

We first assessed whether WLWH differ in terms of pre- and post-vaccination HA stalk antibody Fc-mediated effector functions. To do this, we measured HA stalk-specific ADCP, ADCC reporter activity and ADCD using a chimeric recombinant HA protein with an H6 head, to which humans are generally naïve, and an H1 stalk. TIV administration resulted in significant boosting of HA stalk-specific ADCP in HIV-uninfected women but not in WLWH (**Figure 2A**). Despite this, post-vaccination ADCP activity between the two groups was similar, a consequence of pre-vaccination HA stalk-specific ADCP being significantly higher in WLWH (medians 348.6 vs. 189.7). No ADCP boosting was observed in the placebo groups indicating that these responses were TIV specific (**Supplementary Figure 2**). For ADCC, which was measured using a high throughput FcγRIIIa activation assay, previously shown to correlate with NK degranulation assays (40), there was no significant boosting in either HIV-uninfected women or WLWH (**Figure 2B**). However, like ADCP, pre-vaccination ADCC was significantly higher in the WLWH (medians 154.5 vs. 73.5), and therefore also post-vaccination (medians 170 vs. 83.2). The ADCC activity amongst the vaccinated and placebo groups were similar and remained unchanged since there was no vaccine-mediated boosting of this function (**Supplementary Figure 2**).

To assess Fc-FcγR engagement required for ADCP and ADCC activity, plasma samples were tested for the capacity to bind cH6/1 antigen and cross-link recombinant soluble dimeric FcγRIIa or FcγRIIIa, respectively (39). No boosting in FcγRIIa and FcγRIIIa binding was observed in placebo recipients (**Supplementary Figure 2**). However, as observed in the cell-based functional and reporter assays, FcγRIIa binding was boosted by TIV in HIV-uninfected women but not WLWH, whereas for FcγRIIIa binding, no boosting was observed in either group (**Supplementary Figures 3A, B** **)**. Furthermore, strong positive Spearman’s correlations were noted between FcγRIIa binding and ADCP scores (r=0.44; p<0.0001), and between FcγRIIIa binding and ADCC RLUs (r=0.55; p<0.0001) (**Supplementary Figures 3C, D** **)**.

### Seasonal TIV Substantially Boosts ADCD but Women Living With HIV Have Lower Responses Both Pre- and Post-Vaccination

Since ADCD has been shown to confer protection from influenza virus infection (16, 21), we examined this function in WLWH and HIV-uninfected women, before and after TIV vaccination. ADCD, measured using a bead-based flow cytometry assay, was significantly boosted by TIV in all participants irrespective of HIV status (**Figure 2C**). However, ADCD was substantially impaired in WLWH prior to vaccination and post-vaccination remained significantly lower in comparison to the HIV-uninfected women after TIV boosting (medians 16.2 vs. 225). Placebo recipients showed no increase in ADCD activity (**Supplementary Figure 2**).

### The Magnitude of Vaccine-Mediated Boosting of ADCP and ADCD Is Lower in Women Living With HIV

When we compared Fc-mediated functions, we observed differential boosting across the HIV-uninfected women and WLWH for ADCP and ADCD following vaccination (**Figure 2D**). For ADCP the WLWH were not boosted, and the proportion of high responders (individuals that exceeded a 2-fold increase in Fc activity) was significantly lower than in the HIV-uninfected group (17% vs. 54%). Since there was no boosting of ADCC potential, the proportion of high responders was similar between HIV-uninfected women and WLWH (31% vs. 27%). However, for ADCD, in the WLWH the TIV boosting and the proportion of high responders was significantly lower (median fold change 1.8 and high responders 48%) than in the HIV-uninfected women (median fold change 2.7 and high responders 75%). Thus, while ADCD activity was boosted in both groups in response to TIV vaccination, this function was substantially impaired in WLWH prior to vaccination.

### Seasonal TIV Does Not Improve Overall Co-Ordination of the Fc-Mediated Responses, Irrespective of HIV Status

**Figure 3:**
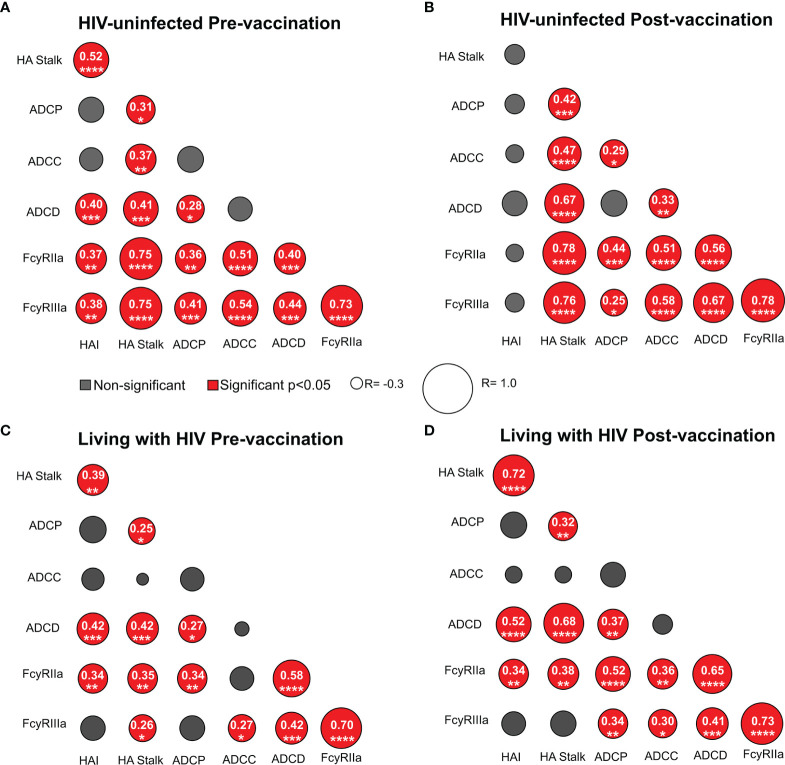
Co-ordination between HAI titers, HA stalk antibodies and HA stalk-specific Fc-mediated functions in response to vaccination. Spearman correlations of Fc effector function and titers in **(A)** HIV-uninfected pre-vaccination, **(B)** HIV-uninfected post-vaccination, **(C)** WLWH pre-vaccination and **(D)** WLWH post-vaccination groups. The asterisks indicate the following statistical significance, ****p < 0.0001; ***p < 0.001; **p < 0.01; *p < 0.05; ns,not significant. Significant correlations are displayed in red. Non-significant correlations are in grey. The size of the circle is proportional to the Spearman correlation coefficients -0.3 been the smallest and 1 been the largest, indicated inside of circles for correlations that were significant.

Co-ordinated antibody responses elicited by vaccines against other diseases have been associated with increased efficacy (41). We therefore assessed the correlations between HAI titers, stalk-specific responses and Fc-mediated effector functions at baseline and after TIV vaccination and compared these in HIV-uninfected women and WLWH. Prior to vaccination, there was a significant correlation between HAI and HA stalk titers in HIV-uninfected women, but the significant boosting of HAI antibodies resulted in loss of this correlation post-vaccination (**Figures 3A, B** **)**. In contrast in WLWH, while there was a strong correlation between HA stalk and HAI pre-vaccination (**Figure 3C**), HA stalk titers were boosted simultaneously with HAI titers, as previously reported (32) and remained significantly correlated (**Figure 3D**). In the HIV-uninfected women, ADCP, ADCC, ADCD and FcγRIIa and FcγRIIIa binding were significantly associated with the HA stalk titers before vaccination, and these associations improved slightly following vaccination (**Figures 3A, B** **)**. In WLWH, ADCP, ADCD and FcγRIIa and FcγRIIIa binding were associated with HA stalk titers pre-vaccination, with the FcγRIIIa binding been the only association to not improve, after TIV vaccination (**Figures 3C, D** **)**. We also observed varying relationships amongst the Fc-mediated effector functions. In the HIV-uninfected women, we observed a significant correlation between ADCP and ADCD pre-vaccination, but not post-vaccination. Instead, after vaccination we observed a correlation between ADCC potential and both ADCP and ADCD. The FcγRIIa and FcγRIIIa binding correlated with all the Fc functions both pre- and post-vaccination in the HIV-uninfected women. In contrast, in WLWH the existing association between ADCD and ADCP prior to vaccination was strengthened post-vaccination. There was also an in increase in the FcγRIIa and FcγRIIIa binding associations with all the Fc functions, following vaccination in this group. Therefore, vaccination resulted in nuanced improvements of particular functional relationships that differed between WLWH and HIV-uninfected women, but overall co-ordination of antibody responses and Fc-mediated functions was not significantly improved.

### Stalk-Specific Fc Polyfunctionality of Women Living With HIV Is Not Improved by Vaccination and Is Not Driven by H1 Stalk Antibody Titer

**Figure 4:**
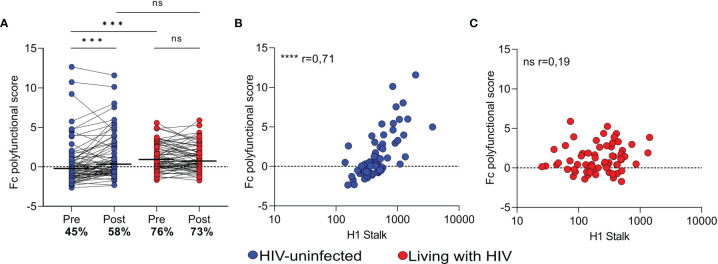
HA stalk-specific Fc polyfunctionality scores of Fc-mediated functions amongst vaccinated women. **(A)** Fc polyfunctionality (determined by the addition of individual standardized Fc function scores), prior to and following vaccination. Dots above 0 indicate Fc polyfunctional individuals, while those below indicate poor Fc polyfunctionality below the mean. The proportion (%) of individuals with Fc polyfunctionality is indicated on the plot. Spearman´s correlation between the Fc polyfunctionality score and HA stalk response in **(B)** HIV-uninfected women and **(C)** WLWH. Wilcoxon matched-pairs signed rank test was used to compare pre-vaccination and post-vaccination polyfunctionality scores within groups. Mann Whitney U test was used to compare polyfunctionality scores between the vaccine groups. Significant associations are shown as ****p < 0.0001; ***p < 0.001; ns,not significant.

Fc polyfunctionality has been shown to be associated with protective immunity and broader responses in other diseases (41–44). We calculated HA stalk-specific Fc polyfunctionality scores by summing the z-scores of ADCP, ADCC and ADCD for each of the participants. Prior to vaccination, WLWH showed significantly higher Fc polyfunctionality directed at the HA stalk than HIV-uninfected women, as a result of their baseline higher ADCC and ADCP responses (medians 0.89 vs. -0.26) (**Figure 4A**). The proportion of participants with high HA stalk-specific Fc polyfunctionality (the sum of z-scores greater than 0) was also higher for WLWH (76%) than for HIV-uninfected group (45%). Following vaccination, HA stalk-specific Fc polyfunctionality in the HIV-uninfected women significantly increased, reaching the same levels observed in WLWH at baseline. In contrast, WLWH did not show improvement of overall HA stalk-specific Fc-mediated functionality following vaccination.

We next assessed the influence of H1 stalk titer on stalk- specific Fc polyfunctionality after vaccination. In the HIV-uninfected group there was a strong correlation between the HA stalk antibody titers and HA stalk- specific Fc polyfunctionality (r=0,71; p<0.0001) (**Figure 4B**). However, in WLWH we observed no correlation between H1 stalk titers and HA stalk-specific Fc polyfunctionality (r=0,19; p=0.26) (**Figure 4C**). The lack of association in WLWH suggests that in this group, the potency of the Fc–mediated functions is mediated through qualitatively different HA stalk-specific antibodies to those occurring in HIV-uninfected women. Overall, these data indicate a fundamentally different humoral response in WLWH in response to vaccination.

## Discussion

HA stalk-specific Fc effector functions have an important complementary role to neutralization in protection against influenza virus infection (45, 46). Therefore, understanding their role in vaccination of high-risk groups, such as PLWH, is crucial but have not been previously studied. In the cohort described here, we observed lower HAI titers and lower HA stalk-specific titers in WLWH than in HIV-uninfected women in response to seasonal influenza vaccination, as reported in our previous studies (6, 32). Despite these lower antibody titers, the vaccine efficacy was higher in WLWH (6). Here, we examined differences in HA stalk-specific Fc-mediated functions between pregnant women living with or without HIV in response to seasonal influenza vaccination in a cohort where the HA stalk antibody titers were previously shown to be protective (32). We observed significant differences in responses; WLWH showed only HA stalk-specific ADCD boosting, whereas in HIV-uninfected women both ADCP and ADCD were significantly enhanced. We also assessed the association between Fc effector functions post-vaccination and found that ADCC or FcγRIIIa activation was coordinated with ADCP and ADCD in HIV-uninfected women but in WLWH only ADCP and ADCD showed improved correlation following vaccination. Furthermore, in HIV-uninfected women overall Fc polyfunctionality was improved following vaccination and was driven by HA stalk antibody titers. In contrast, in WLWH the higher Fc polyfunctionality was not associated with the titers of the HA stalk-specific antibodies. Overall, HA stalk-specific Fc functions were differentially mediated in WLWH in response to seasonal influenza vaccination, despite similar boosting of HA stalk antibodies.

Although the WLWH and HIV-uninfected groups were matched for age, there were substantial differences in their baseline responses, prior to vaccination. Both ADCP and ADCC potential were higher in WLWH pre-vaccination. This may be due to increased previous exposures to influenza virus infections in WLWH prior to vaccination, which may have broadened the HA stalk-specific Fc-mediated functions (6, 45). Multiple exposures to divergent influenza virus strains has been shown to prime HA-specific antibodies with the ability to mediate ADCP and ADCC (47). Several studies have provided evidence of this concept, with H7N9 infection and vaccination of healthy people, inducing cross-reactive, ADCC mediating HA stalk antibodies (13, 18, 48). In addition, the WLWH were on ART and this may have allowed for the partial restoration of past HA stalk-specific Fc functionality and together with the vaccine, provided sufficient immunity against influenza virus infections (22).

In this trial, irrespective of HIV status, HA stalk-specific ADCC (measured either through a reporter assay or binding to dimeric FcγRIIIa) was not boosted following vaccination. TIV has elicited variable ADCC responses in humans, with similar findings to ours reported in healthy individuals (49–51), but moderate ADCC boosting has been reported in older adults and PLWH (22, 24, 52). The lack of boosting of ADCC potential in our study, compared to others, may be a consequence of high pre-existing levels of ADCC responses from prior infections in both groups. These responses may be at a “ceiling” and cannot be further significantly boosted following vaccination. The reporter assay that we use in this study has been shown to correlate with traditionally used CD107a NK degranulation assays (40) and is therefore unlikely the reason for the observed lack of boosting.

We observed differences in functions that were boosted in response to vaccination by HIV status. Specifically, ADCP and binding to dimeric FcγRIIa were only boosted in HIV-uninfected women, similar to previous studies (22). However, WLWH had ADCP levels prior to vaccination that exceeded that of HIV-uninfected women. Similarly, a weaker ADCP vaccine response directed at HA has been previously associated with higher baseline levels, similar to those observed here in HIV-uninfected women (23). Post-vaccination ADCP levels in HIV-uninfected women did not exceed the levels in the WLWH, indicating a maximum threshold in HA stalk ADCP activity. In contrast, ADCD was boosted regardless of HIV status, but was significantly lower in the WLWH both pre and post-vaccination suggesting that the ability of antibodies to perform this function was impaired in this group. Despite similar increasing of HA stalk antibody boosting in response to vaccination there were distinct differences in the ability to boost stalk-specific Fc responses associated with HIV co-infection. Overall, these differences show distinct Fc function between WLWH and HIV-uninfected women in response to vaccination.

Vaccination that induces coordinated Fc effector responses has been associated with efficacy in other infectious diseases (41). We observed a loss in the association between HA stalk titers and HAI activity post-vaccination in the HIV-uninfected group but it was strengthened in the WLWH group, as shown previously (32). While the simultaneous elicitation of both head and stalk directed antibodies in WLWH post-vaccination may indicate that the antibody functions behave in a synergistic manner in this group, how HA stalk-specific Fc effector function is affected by broad HAI antibodies was not investigated in this study. This was also observed through the Fc-mediated functions correlating with both HAI and HA stalk titers in WLWH, whilst the Fc effector functions and FcγR binding did not correlate at all with HAI post-vaccination in HIV-uninfected women (previously shown for ADCD pre-vaccination). This, coupled with the observation that HA stalk antibody titer alone did not correlate with overall Fc polyfunctionality in WLWH, suggests that the quality of the Fc responses may have been impacted by both HAI and HA stalk responses in this group which requires further investigation. In addition, it is possible that modulators of Fc effector function such as isotype or glycosylation may differ as a result of HIV infection (53–55). These differences may drive polyfunctionality more robustly than titer in WLWH. This further highlights the differences between WLWH and HIV-uninfected women HA stalk mediated Fc effector functions, of which the differences in response to seasonal influenza vaccination may explain the elevated protection observed for WLWH.

Chimeric hemagglutinin-based vaccines aimed at eliciting cross-reactive HA stalk antibodies have the potential to be developed as universal vaccines. Correlates of protection for this approach should include HA stalk-specific Fc-mediated functions (56). In this cohort, levels of HA stalk antibodies were protective however only ADCP and ADCD were boosted in response to the vaccine in HIV-uninfected women and only ADCD in WLWH, suggesting that these antibody functions may be more important than ADCC in protection. Future studies should determine whether the higher levels of HA stalk-specific ADCP and ADCC potential in the WLWH were enough to confer protection against influenza virus infection or disease severity. In summary, we showed that although HIV-uninfected women exhibited significantly improved Fc polyfunctionality post-vaccination that was driven by HA stalk titer, WLWH mounted fundamentally different HAI and HA stalk coordinated responses, likely impacted by high baseline HA stalk-specific Fc effector function. Our study highlights the need to include, in future clinical trials, high-risk groups who may have different responses.

## Data Availability Statement

The original contributions presented in the study are included in the article/**Supplementary Material**. Further inquiries can be directed to the corresponding author.

## Ethics Statement

The studies involving human participants were reviewed and approved by Human Research Ethics Committee of the University of the Witwatersrand. The patients/participants provided their written informed consent to participate in this study.

## Author Contributions

BM, PM, and SR conceptualized the study. BM performed experiments, analyzed data, generated the figures and wrote the manuscript. ND performed H1 stalk ELISAs. MN and SM established the trials, collected and provided participant samples and the HAI data. FK provided the chimeric hemagglutinin protein (cH6/1). PM and SR assisted in data interpretation, reviewed and edited the manuscript and supervised the research. All authors contributed to the manuscript and approved the submitted version.

## Funding

This sub-study is funded by the African Leadership in Vaccinology Expertise (ALIVE) research grant of the University of the Witwatersrand. BM is a recipient of bursaries from the South African National Research Foundation, the Poliomyelitis Research Foundation (grant 20/36) and the University of the Witwatersrand postgraduate merit award. PM is supported by the South African Research Chairs Initiative of the Department of Science and Innovation and National Research Foundation of South Africa, the SA Medical Research Council SHIP program (grant 98341), the Centre for the AIDS Program of Research (CAPRISA). SR is a L’Oreal/UNESCO Women in Science South Africa Young Talents awardee. The funders were not involved in the study design, collection, analysis, interpretation of data, the writing of this article or the decision to submit it for publication. Related research by the authors is conducted as part of the DST-NRF Centre of Excellence in HIV Prevention, which is supported by the Department of Science and Technology and the National Research Foundation. Generation of reagents in the Krammer laboratory was supported by Centers of Excellence for influenza Research and Response (75N93021C00014) and Collaborative Influenza Vaccine Innovation Centers (75N93019C00051).

## Conflict of Interest

FK reports royalties (Avimex), consulting fees (Pfizer, Seqirus, Third Rock Ventures and Avimex), and payment for academic lectures during the past two years. FK is also named as inventor on IP filed by the Icahn School of Medicine at Mount Sinai for influenza virus vaccines and therapeutics, SARS-CoV-2 vaccines and SARS-CoV-2 serological assays.

The remaining authors declare that the research was conducted in the absence of any commercial or financial relationships that could be construed as a potential conflict of interest.

## Publisher’s Note

All claims expressed in this article are solely those of the authors and do not necessarily represent those of their affiliated organizations, or those of the publisher, the editors and the reviewers. Any product that may be evaluated in this article, or claim that may be made by its manufacturer, is not guaranteed or endorsed by the publisher.
