# Supplementary material for: Influenza Vaccination Results in Differential Hemagglutinin Stalk-Specific Fc-Mediated Functions in Individuals Living With or Without HIV
Source: Front Immunol. 2022 Apr 19;13:873191. doi: 10.3389/fimmu.2022.873191 (PMC9062095; doi:10.3389/fimmu.2022.873191)
Supplement: Supplementary file 1 [file DataSheet_1.docx]

**Supplementary Material**

Supplementary Table 1: Age and days between visits of vaccinated women.

| **Vaccinated individuals** | **HIV-uninfected**  **n=67** | **Living with HIV**  **n=66** | ***P Value*** |
| --- | --- | --- | --- |
| **Median age; years [IQR]** | 25 [18-39] | 27 [18-38] | ns 0.1406 |
| **Days between pre- and post- vaccination visit; Median [IQR]** | 31 [28-33] | 30 [28-31] | ns 0.3427 |

Mann Whitney U tests used to compare between the vaccine groups, ns=not significant.


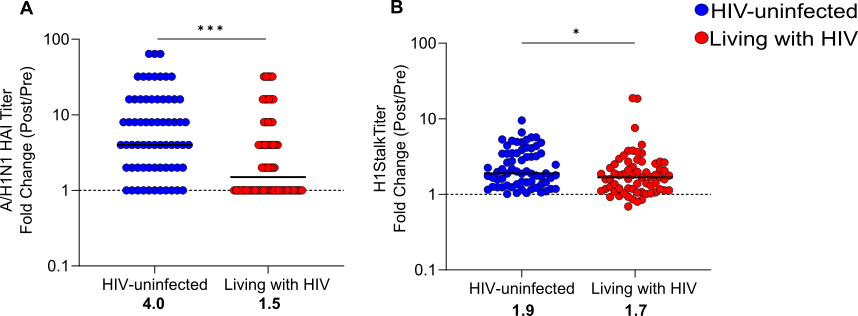


Supplementary Figure 1: Fold changes in A/H1N1 HAI and H1 stalk antibody responses amongst vaccinated women. (A) The fold increases 1-month post-vaccination of hemagglutination inhibition (HAI) titers against A/H1N1 and (B) H1 stalk titers by ELISA, with HIV-uninfected participants (n=67), shown in blue and participants living with HIV (n=66), shown in red. The lines represent the median. Mann Whitney U test used to compare responses between vaccine groups. Significant associations shown as ***p<0.001, *p<0.05.


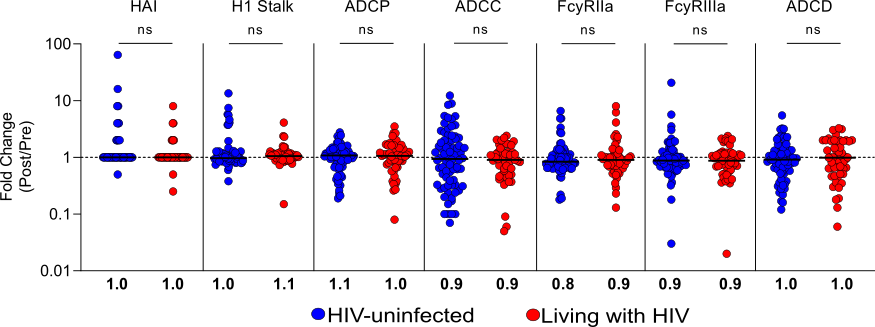


**Supplementary Figure 2: Fold changes in antibody responses, Fc binding and function amongst placebo groups.** HIV-uninfected participants (n=74), shown in blue and participants living with HIV (n=53), shown in red. No significant difference in fold change, indicated below plot, across HAI titers, H1 stalk titers, dimeric FcγR binding and Fc-mediated functions, ADCP, ADCC and ADCD. The lines represent the median. Mann Whitney U test compared placebo groups, ns=not significant**.**


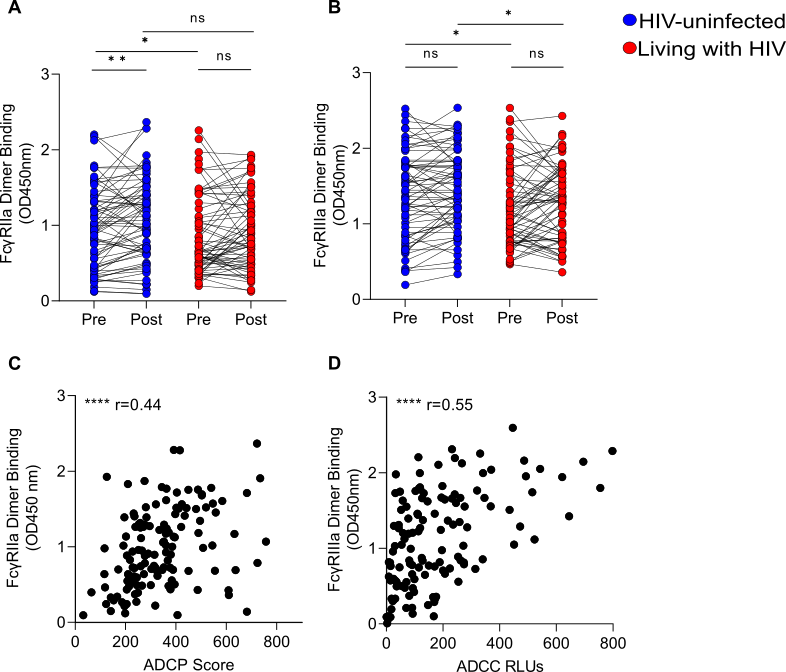


**Supplementary Figure 3: Dimeric Fc gamma receptor (FcγR) binding correlates with HA stalk ADCP and ADCC.** Pre-vaccination and 1-month post-vaccination (A) FcγRIIa dimer binding and (B) FcγRIIIa dimer binding. HIV-uninfected participants (n=67), shown in blue and participants living with HIV (n=66), shown in red. Post-vaccination Spearman’s correlations of (C) FcγRIIa dimer binding by ELISA and ADCP activity and (D) FcγRIIIa dimer binding by ELISA and ADCC activity. Wilcoxon matched-pairs signed rank tests for pre- and post-vaccination comparisons. Mann Whitney U test compared vaccine groups. Significant associations shown as ****p<0.0001, **p< 0.01, *p<0.05, ns=not significant.
